# Infarctus splénique révélant une endocardite infectieuse chez une femme enceinte: à propos d’un cas et brève revue de littérature

**DOI:** 10.11604/pamj.2018.30.184.14262

**Published:** 2018-06-27

**Authors:** Chtioui Mamoun, Fagouri Houda

**Affiliations:** 1 Centre Médico-chirurgical, Université Ibn Zohr, Agadir, Maroc; 2 Service de Gynécologie-Obstétrique, Hôpital Militaire d’Instruction, Rabat, Maroc

**Keywords:** infarctus splénique, endocardite infectieuse, grossesse, accident vasculaire cérébral ischémique, Splenic infarction, infectious endocarditis, pregnancy, ischemic stroke, antibiotic

## Abstract

Le diagnostic d’infarctus splénique est rarement évoqué chez la femme enceinte. L’incidence actuelle de ces atteintes notamment au cours des endocardites infectieuses ainsi que les moyens diagnostiques utilisés sont mal précisés dans la littérature. Une femme de 26 ans, sans antécédent particulier ni facteur de risque cardio-vasculaire, au terme de 14 semaines d’aménorrhée, consulte en urgence, pour un syndrome fébrile évoluant depuis 10 jours et l’apparition récente de douleur abdominale au niveau de l’hypochondre gauche. L’examen retrouve une fièvre à 39,5°C, une sensibilité de l’hypochondre gauche, et des panaris au niveau de la paume de la main gauche et de la plante du pied gauche. L’examen gynécologique était strictement normal. Devant ce tableau, une échographie abdominale réalisée retrouve une image anéchogène médiosplénique à sommet hilaire et à bords périphériques en faveur d’un infarctus splénique. Dans le cadre du bilan étiologique, une échocardiographie réalisée révèle une greffe oslérienne sur la valve mitrale qui est épaissie et remaniée, avec une végétation au niveau de la grande valve et une IM grade II. Des hémocultures ont été réalisées lors des pics fébriles qui étaient revenues positives au staphylocoque doré. L’évolution était marquée par la survenue d’un accident vasculaire cérébral ischémique étendu et aggravation de son état neurologique, conduisant au décès après plusieurs embolies systémiques. L’atteinte splénique chez une femme enceinte est très rare. Toutefois, devant une douleur abdominale aigue de l’hypochondre gauche, l’examen clinique et radiologique ne doit pas omettre l’exploration de la rate. Dans le cas présent, la douleur de l’hypochondre gauche associée à une fièvre et des faux panaris d’Osler s’est révélée être un infarctus splénique en rapport avec une endocardite infectieuse. Une antibiothérapie probabiliste de première intention lors de l’endocardite infectieuse est justifiée et devra être secondairement adaptée aux résultats bactériologiques. Rare, l’infarctus splénique peut avoir des conséquences sévères telles que des abcès ou une rupture, ce qui doit appeler à une certaine vigilance.

## Abstract (en)

The diagnosis of splenic infarction is rarely reported in pregnant women. Current incidence of splenic infarction, especially during infectious endocardites as well as diagnostic methods used are poorly specified in the literature. We here report the case of a 26-year old woman with no particular previous history or cardiovascular risk factor who, at the end 14 weeks of amenorrhea, presented to the Emergency Department with febrile syndrome evolving over 10 days and abdominal pain of recent onset at the level of the left hypochondre. Clinical examination showed febrile patient with a temperature of 39.5°C, tenderness of the left hypochondre and panaritium at the level of the palm of the left hand and of the sole of the foot. Gynecological examination was strictly normal. Given this clinical picture, abdominal ultrasound showed mediosplenic anechoic area with hilar apex and with peripheral edges, suggesting splenic infarct. Etiological assessment included echocardiography showing thickened and remodeled oslerian graft on the mitral valve with large valve vegetation and MI grade II. Blood cultures were performed during the febrile peaks and were positive for golden staph. Patient’s evolution was marked by the occurrence of large ischemic stroke and worsening of neurological condition, leading to death after several systemic emboli. Splenic infarction in a pregnant woman is very rare. However, clinical and radiological examination of the spleen must be performed in patients with acute abdominal pain of the left hypochondre. In the present case, pain of the left hypochondre associated with fever and Osler’s false whitlow was found to be splenic infarction associated with infectious endocarditis. Probabilistic antibiotic therapy as first-line therapy is justified during infective endocarditis and should be secondarily adapted to the bacteriological results. Although rare, splenic infarction can have severe consequences such as abscesses or rupture, which must encourage vigilance.

## Introduction

Les atteintes spléniques au cours des endocardites infectieuses correspondent à des infarctus ou à des abcès; la physiopathologie suggère un continuum entre ces deux types de lésion. Le diagnostic d’infarctus splénique est rarement évoqué chez la femme enceinte. L'incidence actuelle de ces atteintes notamment au cours des endocardites infectieuses ainsi que les moyens diagnostiques utilisés sont mal précisés dans la littérature.

## Patient et observation

**Figure 1:**
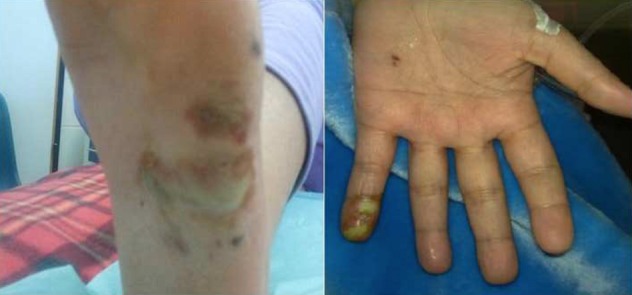
Panaris au niveau de la paume de la main gauche et de la plante du pied

**Figure 2:**
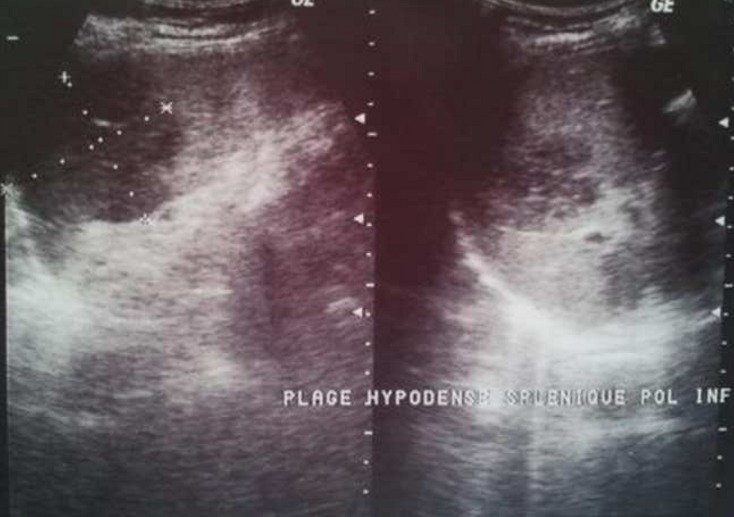
Image anéchogène médiosplénique à sommet hilaire et à bords périphériques en faveur d’un infarctus splénique

Nous rapportons l’observation d’une femme de 26 ans, sans antécédent particulier ni facteur de risque cardio-vasculaire, au terme de 14 semaines d’aménorrhée, consulte en urgence, pour un syndrome fébrile évoluant depuis 10 jours et l’apparition récente de douleur abdominale au niveau de l’hypochondre gauche. L’examen trouve une fièvre à 39,5°C, une sensibilité de l’hypochondre gauche, et des panaris au niveau de la paume de la main gauche et de la plante du pied gauche (Figure 1) ainsi que l’examen cardio-vasculaire trouve un souffle d’IM 2/6. L’examen gynécologique était strictement normal. Devant ce tableau, une échographie abdominale réalisée retrouve une image anéchogène médiosplénique à sommet hilaire et à bords périphériques en faveur d’un infarctus splénique (Figure 2). Dans le cadre du bilan étiologique, une échocardiographie trans-thoracique réalisée révèle une greffe oslérienne (confirmé sur l’échocardiographie trans-oesophagienne) sur la valve mitrale qui est épaissie et remaniée, avec une végétation au niveau de la grande valve et une IM grade II. Des hémocultures ont été réalisées lors des pics fébriles qui étaient revenues positives au staphylocoque doré. La porte d’entrée n’a pas été identifiée. La conduite à tenir initiale fut de réhydrater la patiente avec administration d’antipyrétique et d’une antibiothérapie probabiliste à base de ceftriaxone, qui a été changée par la suite après les résultats de l’hémoculture: bi-antibiothérapie adaptée à la grossesse (flucloxacilline et gentamicine). L’évolution était marquée par la survenue d’un accident vasculaire cérébral ischémique étendu et aggravation de son état neurologique. Toutefois elle a présenté plusieurs complications fatales: une endophtalmie gauche à J+38 de son hospitalisation mise sous traitement antibiotique locale, des infections respiratoires à répétition avec dépendance au respirateur, elle a été trachéotomisée à J+54, elle a avorté à 24 S.A; ensuite à J+100 de son hospitalisation, elle a présenté une thrombose de l’artère fémorale gauche qui a été opérée en urgence avec réalisation d’une embolectomie avec un cathéter à ballonnet de Fogartiy. Ensuite elle a présenté une deuxième ischémie aigue du membre gauche, où une amputation a été nécessaire, et finalement la patiente est décédée suite à un choc septique non jugulé par les drogues vasoactives et les antibiotiques à J+135 de son hospitalisation.

## Discussion

Très souvent, dans l’EI, il se forme des emboles aseptiques et rarement septiques à partir des valves infectées. Ces emboles, viennent obstruer l’artère splénique et provoquent un infarcissement, qui peut se surinfecter par la suite donnant un abcès splénique et même se compliquer d’une rupture splénique [1]. L’atteinte splénique chez une femme enceinte est très rare. La rareté de cette association d’une part et l’abondance des diagnostics différentiels qui s’imposeront à l’obstétricien d’autre part, font que le diagnostic est souvent tardif. Toutefois, devant une douleur abdominale aiguë de l’hypochondre gauche, l’examen clinique et radiologique ne doit pas omettre l’exploration de la rate. Dans le cas présent, la douleur de l’hypochondre gauche associée à une fièvre et des faux panaris d’Osler s’est révélée être un infarctus splénique en rapport avec une endocardite infectieuse. Le diagnostic différentiel de l’infarctus splénique est l’abcès, le scanner permettrait de mieux les différencier avec une meilleure sensibilité (95%) par rapport à l’échographie et une spécificité de 92% [2] et le seul examen pouvant affirmer le diagnostic est l’aspiration à l’aiguille fine scannoguidée, ces deux derniers sont à éviter chez la femme enceinte vue le risque d’irradiation. Seule une échographie abdominale a été donc réalisée qui a permis de mettre en évidence une image anéchogène triangulaire médiosplénique à sommet hilaire en rapport avec un infarctus splénique. Une antibiothérapie probabiliste de première intention lors de l’endocardite infectieuse est indiquée et devra être secondairement adaptée aux résultats bactériologiques, en tenant compte de la grossesse. Le traitement de l’infarctus splénique est conservateur avec une antalgie, une hydratation et une surveillance. Lors d’un abcès splénique, un drainage percutané peut être discuté, mais lors de la survenue d’une rupture de rate, la splénectomie sera alors réalisée en urgence. En effet, la splénectomie précoce permet d’éviter une mortalité importante rencontrée chez les patients opérés tardivement. La survenue de complications emboliques neurologiques rendent le pronostic plus sombre [3]. Les complications neurologiques surviennent dans un tiers des cas après l’initiation de l’antibiothérapie, le plus souvent dans les deux premières semaines; exceptionnellement, elles sont rapportées plusieurs mois après la fin de l’antibiothérapie. Elles sont inaugurales dans 10 à 20% des cas [4].

## Conclusion

Rare, l’infarctus splénique peut avoir des conséquences sévères telles que des abcès ou une rupture, ce qui doit appeler à une certaine vigilance. L’endocardite infectieuse est l’une des diagnostics à évoquer devant un infarctus de la rate. La difficulté chez la femme enceinte est d’affirmer le diagnostic afin d’évaluer le retentissement fœto-maternel et d’adapter la prise en charge thérapeutique.

## Conflits d’intérêts

Les auteurs ne declarent aucun conflit d'intérêts.

## References

[1] Trouillet JL, Hoen B, Battik R, Michel PL, Canavy I, Brochet E, Wolff M, Selton-Suty C, l'Association pour l'étude et la prévention de l'endocardite infectieuse (1999). Les atteintes spléniques au cours des endocardites infectieuses. Rev de Med Int.

[2] Burnier C, Ribordy-Baudat V, Lamy O (2007). Abcès splénique: étiologie,diagnostic et possibilités thérapeutiques. PRAXIS.

[3] Ceulemans B, Louvain MED (2002). Les ruptures spléniques pathologiques.

[4] Duval X, Laissy J-P, Leport C (2009). Complications neurologiques des endocardites infectieuses: épidémiologie, diagnostic, prise en charge, pronostic. Dossier Thématique.

